# Semaphorin 7A as a potential immune regulator and promising therapeutic target in rheumatoid arthritis

**DOI:** 10.1186/s13075-016-1217-5

**Published:** 2017-01-21

**Authors:** Jianmin Xie, Hao Wang

**Affiliations:** 1 Department of Rheumatology, Second Affiliated Hospital of Nanjing Medical University, Nanjing, People’s Republic of China; 2 Experimental Center of Basic Medicine, Shanghai Medical College, Fudan University, Shanghai, People’s Republic of China

**Keywords:** Rheumatoid arthritis, Semaphorin 7A, ADAM17, Cytokine, T cell

## Abstract

**Background:**

Semaphorin 7A (Sema7A) is expressed by several different classes of lymphoid and myeloid cells and is a potent immunomodulator. We examined the role of Sema7A in modulating cellular immune responses and to provide experimental data validating the therapeutic potential of Sema7A in rheumatoid arthritis (RA).

**Methods:**

Soluble Sema7A (sSema7A) levels in the serum and synovial fluid from patients with RA or osteoarthritis, as well as cytokine secretions, were analyzed with an enzyme-linked immunosorbent assay. The cell surface levels and transcripts of Sema7A were evaluated in T cells and monocytes from patients with RA. The effect of Sema7A on the functions of primary T cells isolated from the peripheral blood of healthy donors was observed. Detection of the activation of the signal mediator focal adhesion kinase was performed by Western blotting. Shedding of sSema7A was evaluated in monocytes. The introduction of anti-Sema7A antibody to mice with collagen-induced arthritis (CIA) was observed in vivo.

**Results:**

Upregulation of sSema7A levels in both the serum and synovial fluid of patients with RA was correlated with disease activity markers. sSema7A markedly increased Th1/Th17 cytokine secretion and induced evident upregulation of T-bet and retinoic acid receptor-related orphan nuclear receptor γt levels in T cells. Cell surface Sema7A was cleaved by a disintegrin and metalloprotease 17 (ADAM17) in monocytes. Interleukin-6 and tumor necrosis factor-α stimulated ADAM17 secretion in synovial macrophages. Blocking of β1-integrin abrogated the Sema7A-mediated cytokine secretion. Treatment with an anti-Sema7A antibody significantly attenuated CIA.

**Conclusions:**

These findings indicate that Sema7A as a potent activator of T cells and monocytes in the immune response contributes to the inflammation and progression of RA, suggesting its therapeutic potential in the treatment of RA.

## Background

Rheumatoid arthritis (RA) is an autoimmune and systemic inflammatory disorder characterized by synovial inflammation, destruction of cartilage and bone, and systemic manifestations [1, 2]. RA synovial inflammation evokes arthritis symptoms and causes articular bone and cartilage destruction. In RA joints, the immune cells infiltrating the synovium include T cells, macrophages, B cells, and dendritic cells [3]. Activated synovial T cells and macrophages secrete various types of proinflammatory cytokines, including interleukin-6 (IL-6), tumor necrosis factor-α (TNF-α), and IL-17 [4, 5]. Among the different inflammatory cells, T-helper cell type 1 (Th1) cells, as potent cytokine producers, are considered to be crucial effector cells in RA [6]. Recently, considerable evidence has been presented, both in humans and in mice, for the importance of Th 17 cells as the main source of IL-17 in the development and progression of autoimmune diseases. However, the role of inflammatory cells in RA has remained elusive.

Semaphorins comprise a large family of transmembrane proteins and secreted proteins that have been described as axon guidance molecules during neuronal development. They are divided into eight classes based on sequence similarities and distinct structural features [7, 8]. Of the eight classes of semaphorins, classes 1 and 2 semaphorins are found in invertebrates, whereas classes 3–7 semaphorins are found in vertebrates and class 8 semaphorins are viral. Plexin-semaphorin-integrin domains, immunoglobulin-like domains, PDZ domains, and/or thrombospondin domains have also been shown to be part of the semaphorin protein structure. Semaphorins were initially described for their role in axon guidance during neurogenesis [9]. It has since been found that semaphorins branch beyond the nervous system to cover an ample overlap of molecular repertoires. Semaphorins are also involved in physiological processes such as organogenesis, immune cell regulation, and vascular growth [10].

Semaphorin 7A (Sema7A) is the only class 7 semaphorin, also known as CD108, with a membrane-associated, glycosylphosphatidylinositol (GPI)-linked semaphorin protein that contains arginine-glycine-aspartate [11], a well-conserved, integrin-binding motif, in its Sema domain. Sema7A has an immune function in both the innate and adaptive immune systems [12]. Previous studies have shown that semaphorins have important roles in immunologic disorders, such as multiple sclerosis, contact hypersensitivity, experimental autoimmune encephalomyelitis, granulomatosis with polyangiitis (Wegener’s), and RA [13, 14]. Recently, on one hand, several studies have shown that Sema7A is a potent stimulator of monocytes inducing the production of proinflammatory cytokines [15]. On the other hand, Sema7A has been shown to function as a regulator of the T-cell immune response by its effects on T-cell proliferation [16]. Notably, Sema7A has been demonstrated to have a clear effect on the migration processes of osteoblasts and osteoclasts in bone remodeling and a distinct role in tumor angiogenesis [17, 18]. Neovascularization, immunologic abnormality, and bone erosion all have crucial roles in the progression of RA [19, 20], suggesting that Sema7A may aggravate RA. However, the pathologic significance of Sema7A in RA remains unclear.

On the basis of these observations, we analyzed the role of secreted Sema7A in the modulation of cellular immunological functions and the pathogenesis of RA. Our findings identified that Sema7A is an attractive activator of T cells and monocytes in the synovial lesions of patients with RA. Furthermore, the elevated levels of soluble semaphorin 7A (sSema7A) were proteolytically shed from the cell surface, and the resultant sSema7A contributed to inflammation, suggesting that an inflammatory activation loop exists in RA synovium. Moreover, we demonstrate that Sema7A mediates its intracellular effects via interactions with β1-integrins and the activation of focal adhesion kinase (FAK). The inhibition of Sema7A attenuated the development of collagen-induced arthritis (CIA). In the present study, we explored the key role of Sema7A in immunoregulation, and we provide experimental data validating the therapeutic potential of Sema7A in RA.

## Methods

### Subjects

Blood samples were collected from 55 patients with RA and 12 with osteoarthritis (OA). Blood samples were also obtained from healthy donors. Synovial tissue and fluid samples were collected during knee joint replacement surgery from patients with RA (*n* =15) and patients with OA (*n* = 10). These patients presented with high levels of C-reactive protein (CRP) (29–138 mg/L, mean 66 mg/L) and rheumatoid factor (RF) (63–432 U/ml, mean 214.8 U/ml). Thirty-three of the 55 patients with RA had a Disease Activity Score in 28 joints (DAS28) ≥3.2 [21]. All patients with RA fulfilled the revised American College of Rheumatology criteria [22]. All cases of OA were classified as grade IV on the basis of clinical and radiological criteria according to the Kellgren-Lawrence grading scale [23]. All of the patients provided written informed consent to participate in this study. The ethics committee of Nanjing Medical University approved this study.

### Cell isolation and culture

Isolation of human peripheral blood mononuclear cells (PBMCs) was performed as described previously [20]. PBMCs were analyzed on a fluorescence-activated cell-sorting (FACS) flow cytometer (BD Biosciences, San Jose, CA, USA) using FlowJo software (FlowJo, Ashland, OR, USA). CD4^+^ T cells and CD14^+^ monocytes were purified from PBMCs by incubating cells with anti-CD4 and anti-CD14 monoclonal antibody (mAb). The following antibodies were used: anti-human CD4 phycoerythrin (PE) (BD Biosciences) and anti-human CD14 PE (BD Biosciences). The purity of isolated CD4^+^ T cells and CD14^+^ monocytes was >95% in all experiments. The cell surface expression of Sema7A in peripheral blood CD4^+^ and CD14^+^ cells was detected by flow cytometric analyses using PE-conjugated anti-human Sema7A (BioLegend, San Diego, CA, USA). THP-1 cells were cultured in RPMI 1640 medium supplemented with 10% heat-inactivated FBS and penicillin-streptomycin.

### Synovial macrophage isolation

After synovial tissues were digested, the samples were filtered using a cell strainer (BioLegend). The adherent synovial cells were harvested. After overnight culture, primary synovial cells were employed for synovial macrophage isolation using a magnetic-activated cell sorting separation column system (Miltenyi Biotec, Bergisch Gladbach, Germany) as previously described [23]. Adherent cells were incubated with CD14-labeled microbeads for 30 minutes at 4 °C, and this mixture was then applied to a magnetic column. The positive fraction (CD14^+^) was used as the synovial macrophage. CD14^+^ cells were incubated for 24 h with 1 mg/ml of IL-6 and 0.1 mg/ml of TNF-α (PeproTech, Rocky Hill, NJ, USA) in Dulbecco’s modified Eagle’s medium containing 10% fetal calf serum. The concentrations of a disintegrin and metalloprotease 17 (ADAM17) and Sema7A in the culture supernatants were also measured by enzyme-linked immunosorbent assay (ELISA).

### Cytokine production assay

For the analysis of cytokine secretions, freshly isolated CD4^+^ T and CD14^+^ cells (2 × 10^5^) from patients with RA were incubated in the presence of Sema7A-Fc fusion protein (10 ng/ml) at the indicated concentrations for 48 h. Cells cultured in the absence of Sema7A (negative control [NC]) or in the presence of heat-denatured Sema7A (DSema7A) were used as an NC or an additional control. DSema7A was produced by incubating Sema7A at 95 °C for 20 minutes. Supernatants from T-cell cultures were then collected and analyzed for cytokine secretion. The concentrations of human TNF-α, IL-6, interferon-γ (IFN-γ), IL-22, and IL-17 in the culture supernatants were determined using ELISA kits for each cytokine (R&D Systems, Minneapolis, MN, USA). For the blocking experiments, the CD14^+^ cells were cocultured with 10 ng/ml of Sema7A and 10 ng/ml of anti-Sema7A antibody, anti-β1-integrin antibody, or anti-plexin C1 antibody (BioLegend), or with immunoglobulin G (IgG) as a control, for 36 h. The levels of IL-6 and TNF-α in the supernatants were evaluated by ELISA.

sSema7A levels in patients’ serum and synovial fluid samples from and in the cell culture supernatants were detected using an ELISA kit (R&D Systems). The levels of ADAM17 in the serum and synovial fluid samples were also determined using an ELISA kit (MyBioSource, San Diego, CA, USA).

Human Sema7A-Fc fusion proteins were produced as previously described [11, 16], and recombinant human IgG1-Fc (R&D Systems) was used as a control. Naturally cleaved sSema7A was affinity-purified, performed as previously described [16].

### Reverse transcription-quantitative polymerase chain reaction

PBMCs or CD4^+^ or CD14^+^ cells (2 × 10^5^) were isolated using a FACS flow cytometer (BD Biosciences). The total RNA was extracted from the cells using TRIzol reagent (Invitrogen, Carlsbad, CA, USA) according to the manufacturer’s instructions. The complementary DNA was synthesized using SuperScript III reverse transcriptase (Invitrogen) according to the manufacturer’s protocol. A LightCycler (Roche, Basel, Switzerland) and a SYBR reverse transcription-polymerase chain reaction (RT-PCR) kit (Toyobo, Osaka, Japan) were used for the quantitative real-time RT-PCR analysis in accordance with the manufacturer’s instructions. The sequences of the primer sets for Sema7A, β1-integrin, and plexin C1, as well as the target sites on the messenger RNA (mRNA) and the PCR product sizes, are shown in Table 1. β-actin was used to normalize the samples. The relative quantification of each gene was determined using LightCycler 480SW software (Roche).

**Table 1:** Primers for human semaphorin 7A, β1-integrin, and plexin C1

Transcript	Forward primer	Reverse primer	Length
Semaphorin7A	TCATCAAAGCCACCATCG	AGCTCACATACAGCTTCCTCC	771
β1-integrin	CAAAGGAACAGCAGAGAAGC	GTGGAAAACACCAGCAGC	537
Plexin C1	AACCATTGCACTGCAAACC	GATTCCATCTTCAAGAATCACG	557

### Western blot analysis

Cultured CD4^+^ T cells from healthy control subjects were stimulated with Sema7A for 3 h. Unstimulated cells or T cells treated with DSema7A were used as controls. The cells were washed twice with ice-cold PBS and lysed in lysis buffer consisting of 25 mM Tris-HCl, pH 7.5, 150 mM NaCl, 1% Triton X-100, 0.1% SDS, and cOmplete Mini protease inhibitor cocktail (Roche). For phosphorylation-specific immunoblot analysis, phosphatase inhibitors (10 mM NaF, 12.5 mM β-glycerophosphate, and 1 mM Na_3_VO_4_) were added to the lysis buffer. Whole-cell lysates were subjected to SDS-PAGE under reducing conditions. Immunoblotting was performed using antibodies specific to retinoic acid receptor-related orphan nuclear receptor γt (RORγt; BioLegend) and T-bet (BD Biosciences).

The CD14^+^ cells from patients with RA were stimulated with 10 ng/ml of recombinant Sema7A or DSema7A for 15 minutes. CD14^+^ cells stimulated with DSema7A were used as negative controls. The activation of FAK was evaluated by immunoblotting using antibodies specific to FAK (Santa Cruz Biotechnology, Dallas, TX, USA), phospho-FAK (Tyr397) (BD Biosciences), and glyceraldehyde 3-phosphate dehydrogenase (Abcam, Cambridge, UK).

### Shedding of sSema7A

The THP-1 cell line and CD14^+^ cells from patients with RA were cultured in RPMI 1640 medium containing 1 μM of BMS-561392 (Bristol-Myers Squibb, New York, NY, USA) or 100 μM of GM6001 (Calbiochem, San Diego, CA, USA) for 7 minutes at room temperature before the THP-1 and CD14^+^ cells were incubated for 12 h with 0.1 mg/ml of TNF-α and 1.0 mg/ml of IL-6. sSema7A concentrations were measured by Western blotting.

### Small interfering RNA experiments

HEK293T cells were transduced with a pcDNA3.1 vector containing the Sema7A sequence. HEK293T cells stably expressing Sema7A (HEK293T_Sema7A) were infected with a lentiviral vector containing short-hairpin RNA (shRNA) specific for *ADAM17* (5′-GCTTGATTCTTTGCTCTCA-3′) or a nonspecific shRNA. The preparation of the lentiviral vector and cell transduction were performed according to previously described protocols [24]. ADAM17 mRNA expression was assayed by RT-PCR 48 h after cell transduction. sSema7A levels in the cell supernatant were determined by Western blot analysis 5 days after the silencing of ADAM-17 expression.

### Induction and assessment of CIA

The CIA model was induced in the mice as previously described [25, 26]. Briefly, DBA/1 mice were obtained from the Shanghai Animal Center (Shanghai, China). Eight-week-old DBA/1 mice were given intradermal injections of 100 μg/mouse of bovine collagen type II (CII) (Cosmo Bio, Tokyo, Japan) emulsified in complete Freund’s adjuvant containing 250 μg/mouse of heat-killed *Mycobacterium tuberculosis* H37Ra (BD Biosciences). Twenty-one days after immunization, the mice were given a booster injection at the base of the tail with 100 μg/mouse of bovine CII. Mice with CIA were given intraperitoneal injections of 12 μg/mouse of anti-Sema7A antibody (AF1835, goat IgG; R&D Systems) (*n* = 8), 9 μg/mouse of anti-Sema7A antibody (*n* = 8), or isotype control antibodies (R&D Systems) on days 1, 10, 20, 30, and 40. Serum was collected on day 50. Serum levels of TNF-α, IL-6, and IL-17 were evaluated by ELISA (R&D Systems).

To evaluate the incidence and severity of arthritis, arthritis was scored by two calibrated examiners via a visual assessment scoring system using a scale of 0–4 per limb as previously described [25, 27]. The severity scores were defined as follows: 0 = no evidence of erythema and swelling; 1 = erythema and mild swelling confined to the midfoot (tarsals) or ankle joint; 2 = erythema and mild swelling extending from the ankle to the foot; 3 = erythema and moderate swelling extending from the ankle to the metatarsal joints; and 4 = erythema and severe swelling encompassing the ankle, foot, and digits. The paws of mice stimulated with anti-Sema7A antibody (*n* = 8), the control antibody (*n* = 8), or no antibody (*n* = 6) were fixed and decalcified. Histologic scoring and the histomorphometric analysis of the paws were performed by an investigator blinded to treatment status.

### Statistical analysis

Statistical analysis of the data was performed using SPSS version 16 software (SPSS, Chicago, IL, USA). Nonparametric Mann-Whitney *U* tests were used to compare two groups, and comparisons between three groups were performed using the Kruskal-Wallis test followed by the Mann-Whitney *U* test. *P* values less than or equal to 0.05 were considered significant. Correlations between clinical parameters and Sema7A were determined using Spearman’s correlation. The data are presented as SDs.

## Results

### Significantly increased levels of sSema7A in patients with RA and correlation with disease activity

Several members of the semaphorin family have been characterized with respect to their function in immunity. To explore the pathologic role of Sema7A in RA, we first detected the serum levels of Sema7A in patients with immunologic and joint-destructive diseases. The serum levels of secreted Sema7A were determined by ELISA in a large cohort of patients with RA (*n* = 55) and patients with OA (*n* = 12) in comparison with healthy individuals (*n* = 60).

**Fig. 1:**
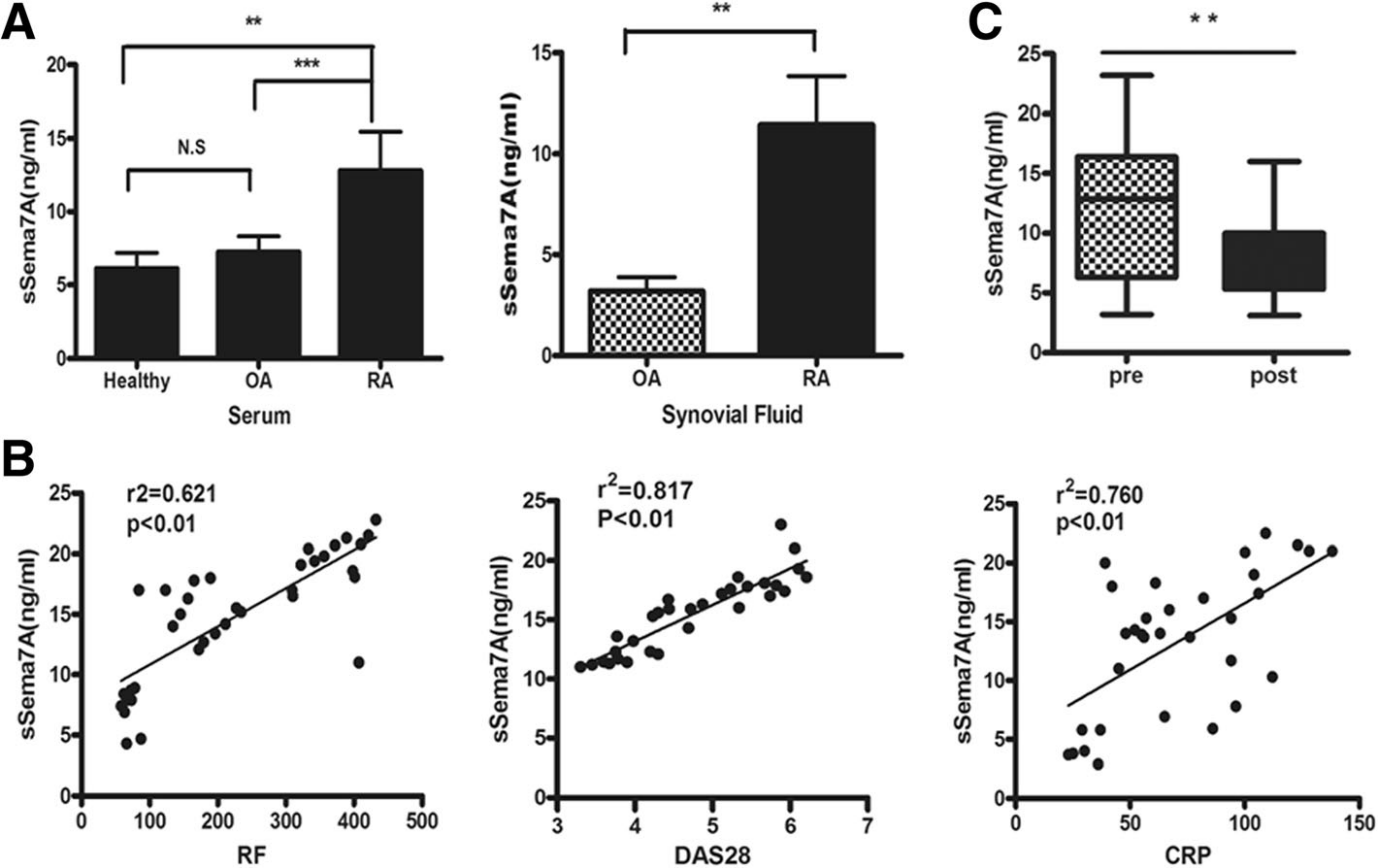
Levels of soluble semaphorin 7A (sSema7A) and correlation with markers of rheumatoid arthritis (RA) disease activity. **a** sSema7A levels in serum and synovial fluid samples from patients with RA and patients with osteoarthritis (OA). **b** Serum sSema7A levels before and after biologic disease-modifying antirheumatic drug treatment in good responders according to the European League Against Rheumatism response criteria. Values in **a** and **b** are mean ± SD. **P < 0.01; ***P < 0.001. *N.S* Not significant. **c** Correlation between serum sSema7A levels with rheumatoid factor (RF) titer, Disease Activity Score in 28 joints (DAS28), and C-reactive protein (CRP) level (*n* = 31 samples)

Interestingly, the levels of serum sSema7A were remarkably increased in patients with RA compared with healthy subjects (mean ± SD 13.6 ± 3.7 ng/ml versus 5.7 ± 2.8 ng/ml; *P* < 0.01). In contrast, serum sSema7A levels were not elevated in patients with OA. We next performed ELISA to determine the Sema7A levels in the synovial fluid of patients with RA and OA. A significantly increased level of Sema7A was detected in fluid from patients with RA (12.3 ± 6.5 ng/ml) compared with that from patients with OA (3.1 ± 2.4 ng/ml) (*P* < 0.01) (Fig. 1a). To investigate the clinical significance of sSema7A, we analyzed the correlations between clinical features and levels of serum sSema7A. Importantly, there was a positive correlation between sSema7A and disease activity markers such as the DAS28 (*r* ^2^ = 0.817, *P* < 0.01), the CRP level (*r* ^2^ = 0.760, *P* < 0.01), and the RF titer (*r* ^2^ = 0.621, *P* < 0.01) (Fig. 1b). In contrast, no relationship between serum sSema7A levels and disease duration, age, or sex were observed (data not shown).

Serum levels of sSema7A were examined before and 6 months after the beginning of biologic disease-modifying antirheumatic drug (DMARD) therapy (31 patients were treated with TNF inhibitors). A significant reduction in serum sSema7A levels after biologic DMARD treatment was observed (mean ± SD 12.6 ± 4.3 ng/ml versus 6.7 ± 2.6 ng/ml; *P* < 0.01) in patients who were good responders according to the European League Against Rheumatism response criteria [28] (Fig. 1c), suggesting the involvement of Sema7A in determining the clinical status of RA.

### Expression of Sema7A and its upregulation of secretion of proinflammatory cytokines in T cells and monocytes/macrophages

**Fig. 2:**
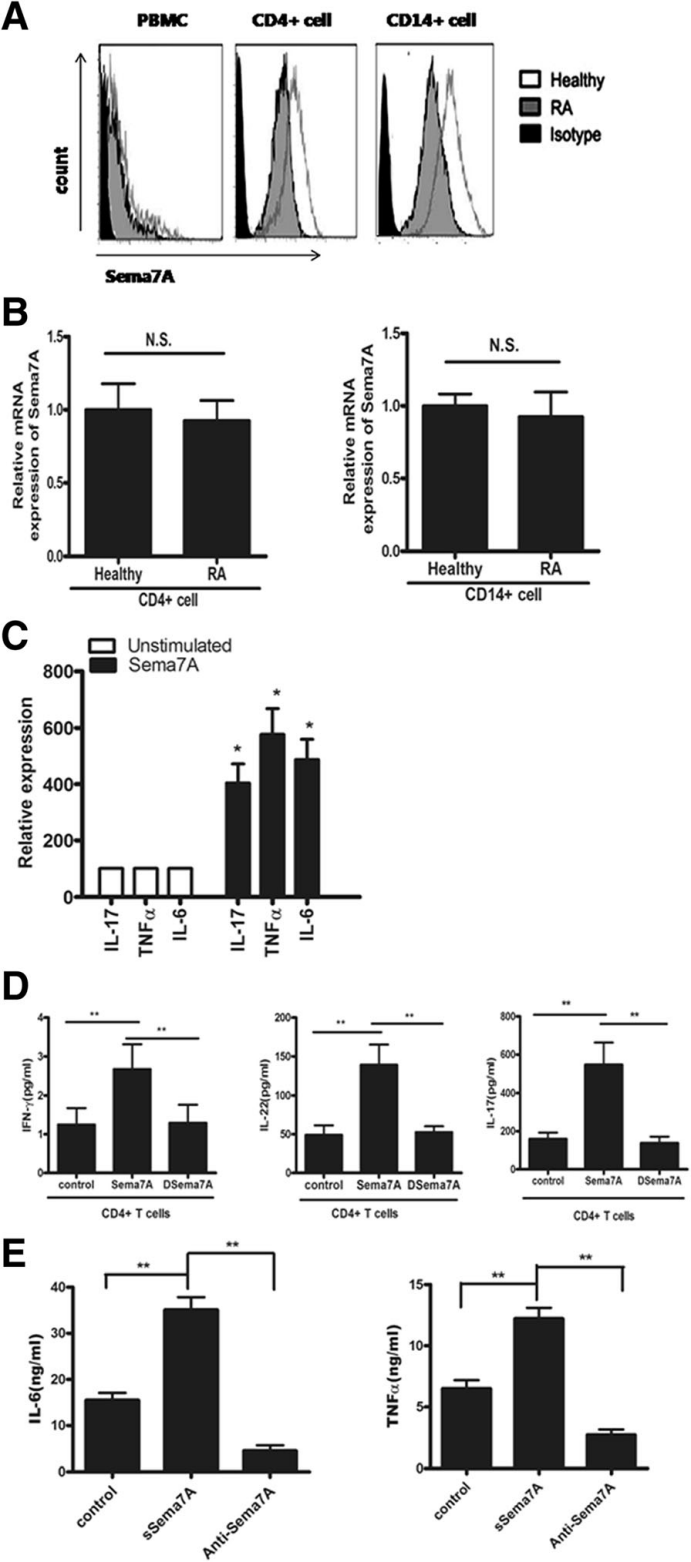
Semaphorin 7A (Sema7A) expression, and inflammatory cytokine synthesis induced by Sema7A in primary human cells. **a** Cell surface expression of Sema7A in peripheral blood CD4^+^ and CD14^+^ cells and peripheral blood mononuclear cells (PBMCs) from patients with rheumatoid arthritis (RA) and healthy volunteer donors. Results shown are representative of findings from nine patients with RA and eight healthy volunteer donors. **b** Expression of messenger RNA (mRNA) for Sema7A in peripheral blood CD4^+^ and CD14^+^ cells. Results shown are from nine patients with RA and eight healthy individuals. *N.S.* Not significant. **c** Cytokine production by PBMCs from healthy donors (*n* = 6 percytokine) after treatment with or without Sema7A (10 ng/ml) for 24 h. Supernatants were analyzed for expression of IL-17, TNF-α, and IL-6 by enzyme-linked immunosorbent assay. Results are shown as the mean of triplicate values ± SD from one of three representative experiments. *P<0.05 versus unstimulated controls. **d** Interferon-γ (IFN-γ), IL-17, and IL-22 synthesis by primary human CD4^+^ T cells from patients with RA after treatment with either Sema7A or heat-denatured Sema7A (DSema7A) (both at 10 ng/ml) for 24 h. Data are shown as the mean of triplicate values ± SD as compared with DSema7A (10 ng/ml) or unstimulated CD4^+^ T cells. **P < 0.01. **e** Tumor necrosis factor-α (TNF-α) and interleukin-6 (IL-6) production by primary human CD14^+^ monocytes from patients with RA after stimulation with naturally cleaved soluble Sema7A (sSema7A) for 48 h with or without anti-Sema7A antibody. Results shown are representative of three independent experiments

Studies suggest that members of the semaphoring family are important players during the immune response because they regulate interactions between T cells and antigen-presenting cells during the primary immune response [29, 30]. To explore whether Sema7A expression by T cells and monocytes differed in patients with RA compared with control subjects, we isolated CD4^+^ cells and CD14^+^ cells and performed flow cytometry. Notably, cell surface Sema7A expression was downregulated on all cells from patients with RA (Fig. 2a). Furthermore, cell surface Sema7A was expressed richly on CD14^+^ and CD4^+^ cells in healthy individuals. In contrast, the expression of the Sema7A transcript was not reduced in CD4^+^ and CD14^+^ cells from patients with RA compared with healthy donors (Fig. 2b), suggesting that the reduction in cell surface Sema7A was due to shedding of Sema7A from the cell surface.

Next, we assessed the effect of Sema7A on the cytokine production of PBMCs from healthy subjects. Culture supernatants were collected after 48 h of incubation and analyzed for secreted cytokines by ELISA. The secretions of IL-6, TNF-α, and IL-17 were significantly increased after stimulation with Sema7A (Fig. 2c). Notably, the in vitro stimulation of CD4^+^ cells with Sema7A led to a significant increase in the secretion of Th17/Tc17 cytokines, including IL-17, IFN-γ, and IL-22, compared with unstimulated cells or cells stimulated with DSema7A (Fig. 2d). These data indicate that sSema7A can strongly increase the production of inflammatory cytokines, in particular Th17/Tc17 and Th1/Tc1, by T cells in vitro.

Previous studies have shown that Sema7A is a potent stimulator of monocytes and induces the production of proinflammatory cytokines. Monocytes and macrophages are critical effector cells of the inflammatory immune response in RA [30, 31]. Next, to examine whether Sema7A serves a crucial role by affecting inflammatory immune functions in RA, we assessed the effect of sSema7A on monocyte IL-6 and TNF-α production among patients with RA in vitro. Treatment with cleaved sSema7A or sSema7A-Fc increased the production of TNF-α and IL-6 by CD14^+^ monocytes/macrophages. Moreover, the anti-Sema7A antibody suppressed IL-6 and TNF-α secretion stimulated by sSema7A (Fig. 2e). These results showed that sSema7A can induce inflammatory cytokine production by monocytes/macrophages, suggesting that Sema7A might have a pathogenic role in RA.

### Role of Sema7A in the expression of T-bet and RORγt by T cells

**Fig. 3:**
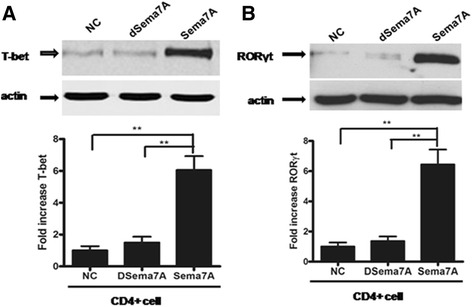
Semaphorin 7A (Sema7A) enhances expression of retinoic acid receptor-related orphan nuclear receptor γt (RORγt) and T-bet transcription factor in CD4^+^ T cells from healthy donors. Freshly isolated T cells (CD4^+^) were stimulated with 10 ng/ml of Sema7A or heat-denatured Sema7A (DSema7A) for 2 h. The expression of (**a**) T-bet and (**b**) RORγt was detected by immunoblotting. Bars show the mean ± SD of three independent experiments. **P < 0.01 versus unstimulated control or DSema7A. *NC* Negative control cells cultured in the absence of Sema7A

Increasing data indicate that the latter types of CD4^+^ cells, such as Th1 and Th17 cells, are key players in the development of RA [6, 31, 32]. Th1 cells are characterized by their expression of the transcription factor T-bet [33]. Th17 cells are a subgroup of helper T cells with the capability to produce high levels of IL-17, which is their main characteristic, along with the expression of the transcription factor RORγt [34]. As mentioned above, T cells stimulated with Sema7A induced the production of cytokines that were relevant to Th1/Tc1 and Th17/Tc17 subclasses. Next, we evaluated the effect of Sema7A stimulation on RORγt and T-bet expression in T cells. After the induction of CD4^+^ cells by Sema7A, we found that Sema7A caused a significant elevation in the expression of both RORγt and T-bet compared with unstimulated cells or cells stimulated with DSema7A (Fig. 3a and b). These data suggested that Sema7A induced CD4^+^ cells to differentiate into Tc1/Tc17 and Th1/Th17 subclasses, respectively.

### Effect of Sema7A receptors on cytokine production

**Fig. 4:**
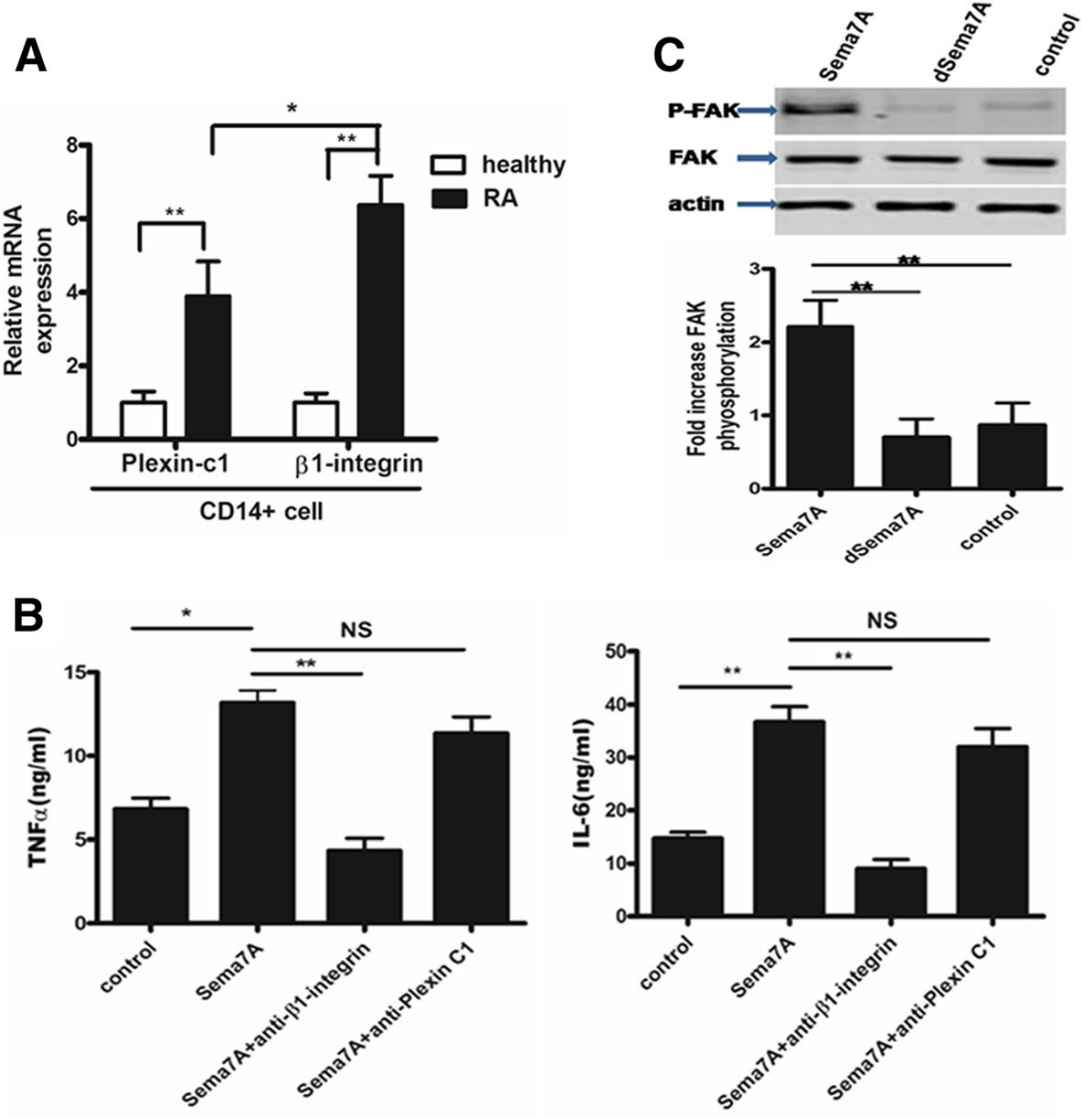
Blocking β1-integrin abrogates semaphorin 7A (Sema7A)-induced cytokine production from CD14^+^ cells in rheumatoid arthritis (RA). **a** Expression of messenger RNA (mRNA) for Sema7A receptors (plexin C1 and β1-integrin) in peripheral blood CD14^+^ cells from patients with RA and from healthy individuals. Results shown are from 14 patients with RA and 12 healthy individuals. Values in a–c are mean ± SEM. *P < 0.05, **P < 0.01. **b** RA CD14^+^ cells were incubated with the anti-β1-integrin monoclonal antibody (mAb) or anti-plexin C1 mAb in the presence of soluble Sema7A. After 48-h cultivation, supernatants were collected for expression of TNF-α and IL-6 by enzyme-linked immunosorbent assay. Results shown are representative of findings from eight patients with RA and six healthy volunteer donors. **c** Phosphorylation of focal adhesion kinase (FAK) in THP-1 and CD14^+^ cells upon exposure to Sema7A. Isolated CD14^+^ cells were exposed to 10 ng/ml of recombinant Sema7A. Cells exposed to heat-inactivated Sema7A (DSema7A) were used as negative controls. Phosphorylation of FAK was evaluated by immunoblot analysis. Activation of FAK was detected after 3 min of incubation with Sema7A. Results shown are representative of three independent experiments. *NS* Not significant

The effects of Sema7A are believed to be mediated via two receptors: plexin C1 and the β1-integrin subunit [35, 36]. To investigate whether β1-integrin and/or plexin C1 is implicated in the stimulatory effect of Sema7A observed in monocytes, we first evaluated the mRNA levels of β1-integrin and plexin C1 by quantitative RT-PCR in CD14^+^ monocytes derived from patients with RA and healthy subjects. We found that the mRNA levels of β1-integrin and plexin C1 were significantly upregulated in patients with RA compared with healthy subjects. Interestingly, the mRNA levels of β1-integrin were significantly higher than the levels of plexin C1 in RA CD14^+^ monocytes (Fig. 4a).

Next, blocking assays were performed with anti-plexin C1 or anti-β1-integrin antibody. The monocytes were stimulated with Sema7A or anti-plexin C1 or anti-β1-integrin antibody, and cytokine secretion was analyzed. Sema7A-induced cytokine secretion was significantly inhibited in the presence of the anti-β1-integrin antibody compared with control cells stimulated with Sema7A in the absence of the anti-β1-integrin antibody. Notably, cytokine secretion by Sema7A was barely inhibited in the presence of the anti-plexin C1 antibody compared with the presence of the anti-β1-integrin antibody (Fig. 4b). These data indicate that Sema7A may induce cytokine secretion from CD14^+^ cells via interactions with β1-integrin. Although it has been proposed that plexin C1 is a Sema7A receptor, the effect of Sema7A on monocytes is independent of plexin C1. These observations further reinforce that β1-integrin may be the major receptor for Sema7A in monocytes from patients with RA.

### Sema7A signaling through β1-integrin activates FAK

Sema7A has been reported to promote axon outgrowth through the activation of non-receptor tyrosine kinase FAK during the embryonic development of neuronal progenitor cells [37]. FAK is a pivotal signal transducer downstream of integrins [38, 39]. Moreover, it is known that signaling through integrins such as β1-integrin activates FAK [39]. To determine whether Sema7A elicits integrin-dependent signaling responses in CD14^+^ monocytes from patients with RA, we measured the level of FAK phosphorylation by performing immunoblot analysis in CD14^+^ monocytes and THP-1 cells stimulated with Sema7A or DSema7A for 1 h. As shown in Fig. 4c, the phosphorylation of FAK was detected after stimulation with Sema7A. In contrast, DSema7A was used as a negative control and did not cause FAK phosphorylation. These data indicate the role of integrin-associated signal transduction machinery in propagating Sema7A signals.

### Cleavage of Sema7A from cell surface

**Fig. 5:**
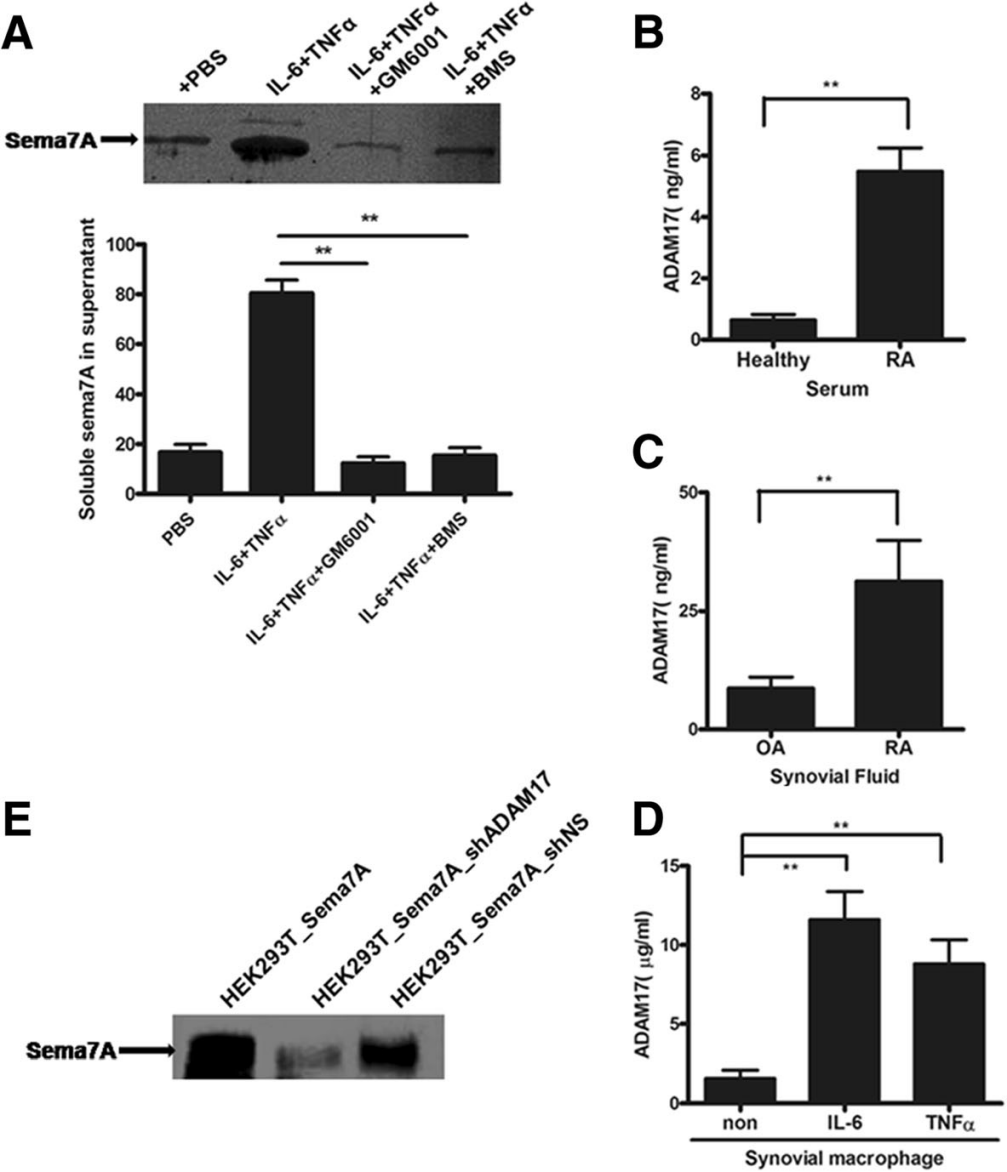
A disintegrin and metalloprotease 17 (ADAM17) contributes to the shedding of semaphorin 7A (Sema7A). **a** Shedding of Sema7A is inhibited by metalloproteinase inhibitors. Western blots show Sema7A in the supernatant of THP-1 and CD14^+^ cells stimulated with tumor necrosis factor-α (TNF-α) and interleukin-6 (IL-6) in the presence of the metalloprotease inhibitor GM6001 or the ADAM17-selective inhibitor BMS-561392 (BMS). Results shown are representative of three independent experiments. **b** Elevated serum ADAM17 levels in 26 patients with rheumatoid arthritis (RA) and 23 healthy donors. **d** Synovial fluid levels of ADAM17 in 11 patients with RA and 8 patients with osteoarthritis (OA). **c** Synovial fluid levels of ADAM17 in 11 patients with RA and 8 patients with osteoarthritis (OA).  **d** ADAM17 protein levels in primary cultures of TNF-α- and IL-6-stimulated synovial macrophages from patients with RA. Data were compiled from three independent experiments. Values are mean ± SEM. **P < 0.01. **e** HEK293T cells stably expressing transmembrane Sema7A (HEK293T_Sema7A) were transduced with vectors encoding an ADAM17-specific short-hairpin RNA (shADAM17) or a nonspecific short-hairpin RNA (shNS). Levels of secreted Sema7A in the cell culture supernatant were analyzed by Western blotting. Results shown are representative of three independent experiments

Previous studies established that Sema7A as a transmembrane protein can produce a soluble form by shedding from the cell surface [40]. The mechanism by which Sema7A is cleaved from the cell surface is not well understood. It is now clear that metalloproteinases are involved in the ectodomain shedding of many important cell surface proteins [41]. Previous studies have established that the cleavage of GPIbα and Sema4D is mediated by ADAM17 [42, 43]. Sema7A is a GPI-anchored member of the semaphorin family [44]. Thus, we determined whether ADAM17 was implicated in the shedding of Sema7A from the monocyte membrane. We incubated Sema7A-expressing cells (THP-1 cells and CD14^+^ cells) with BMS-561392, a potent and selective inhibitor of ADAM17, or GM6001, a general inhibitor of metalloproteinases [45]. GM6001 inhibits Sema7A cleavage by 72% when CD14^+^ cells are activated by IL-6 and TNF-α. The corresponding proportion for BMS-561392 is 65% (Fig. 5a). Thus, the results validated the detection of sSema7A in the shedding analysis and suggest that ADAM17 is probably the shedddase. Consistent with this finding, we measured the enhanced levels of ADAM17 in the serum and synovial fluid of patients with RA (Fig. 5b and c), and we found that TNF-α and IL-6 stimulated the secretion of ADAM17 by monocytes/macrophages detected in the patients with RA in this study (Fig. 5d).

To further investigate the role of ADAM17 in the shedding of Sema7A, we established stable HEK293T cell lines overexpressing ADAM17 or Sema7A. sSema7A was detected in the cell culture supernatants with nonspecific shRNA expression. ADAM17 expression was silenced using a vector encoding for ADAM17-specific shRNA in HEK293T cell lines overexpressing ADAM17 or Sema7A. ADAM17 shRNA resulted in a significant decrease in the levels of sSema7A in the cell culture supernatant of HEK293T cells overexpressing ADAM17 or Sema7A (Fig. 5e). Thus, these results validate the detection of Sema7A in the shedding analysis and suggest that ADAM17 is probably shed in patients with RA.

### Blocking of Sema7A attenuates CIA

**Fig. 6:**
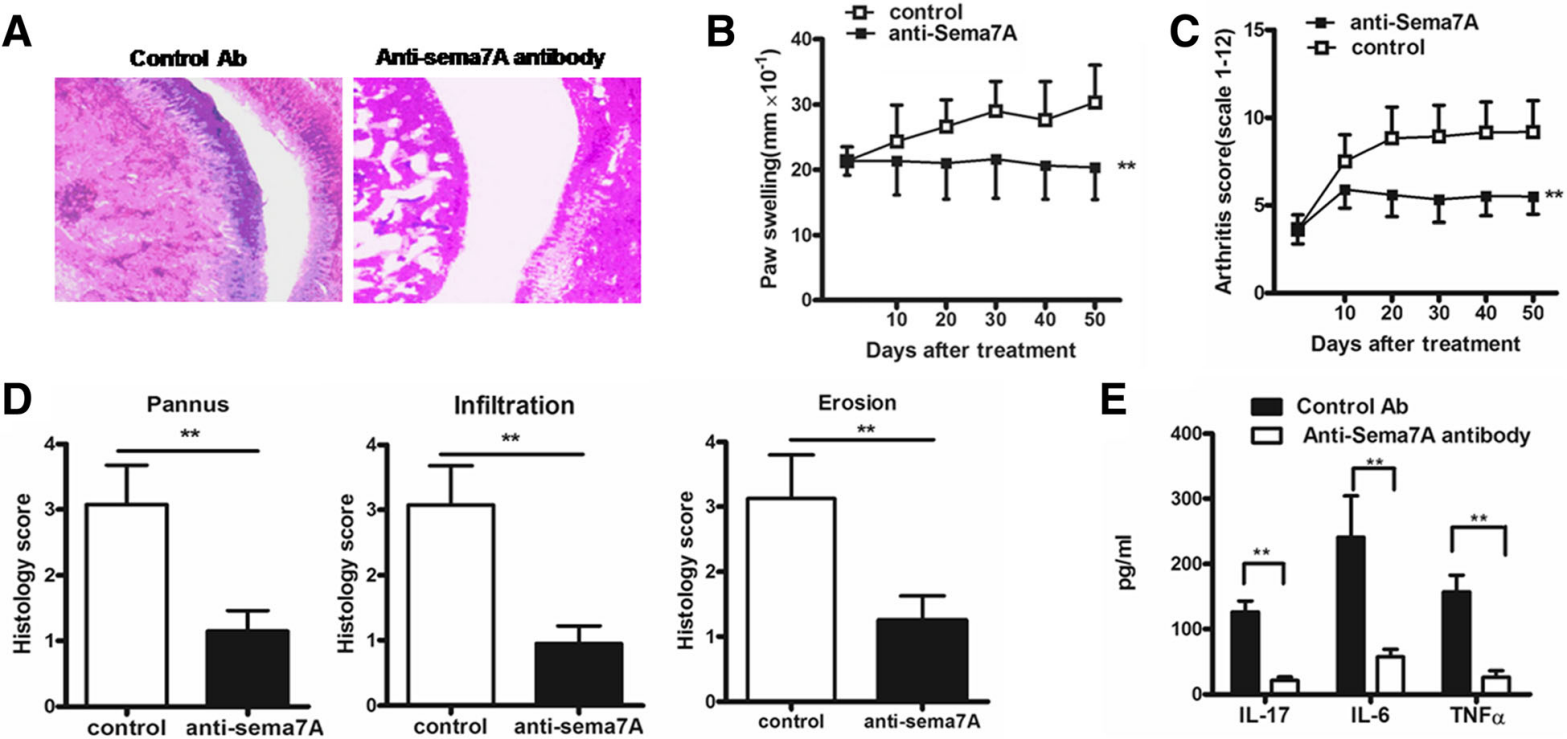
Blocking of semaphorin 7A (Sema7A) attenuates severity of collagen-induced arthritis (CIA) in mice. **a** Histological examination of a mouse ankle joint on day 50 after treatment with anti-Sema7A antibody. Sections were stained with hematoxylin and eosin. Original magnification × 400. **b** and **c** Average arthritis scores and paw swelling of mice with CIA. Mice (*n* = 8 per group) were immunized with collagen type II, randomized, and, at onset of disease (clinical score of 4), anti-Sema7A or control antibody (Ab) (12 μg/mouse) was administered intraperitoneally on days 1, 10, 20, 30, and 40 (*n* = 8 mice per group). Data are representative of three independent experiments. **d** Average histologic scores of paw sections on day 50 (*n* = 8 mice per group). **e** Serum samples were collected for expression of tumor necrosis factor-α (TNF-α), interleukin-17 (IL-17), and IL-6 by enzyme-linked immunosorbent assay on day 50 (*n* = 8 mice per group). Data were compiled from three independent experiments. Values are mean ± SEM. **P < 0.01

Next, we investigated whether the blocking of Sema7A using an anti-Sema7A antibody has the ability to prevent and treat the development of CIA in DBA/1 mice, a surrogate model of human RA. For the CIA treatment studies, mice with established clinical arthritis (an average score of 4) were randomized and given an injection with anti-Sema7A antibody or a control antibody. In the prevention model, blocking Sema7A demonstrated that treatment with anti-Sema7A antibody significantly reduced the development of CIA. Indeed, we observed that the arthritis scores and paw swelling in mice treated with anti-Sema7A antibody were significantly reduced compared with those in control mice (Fig. 6b), and the reduction in the arthritis scores was attenuated in mice treated with half a dose of the antibody. Histologic examination revealed that blocking Sema7A in mice with CIA also reduced disease severity and was accompanied by reduced bone erosion, synovial hyperplasia, and inflammatory infiltration into the joint compartment (Fig. 6a). The histologic scores in the joints of mice treated with anti-Sema7A antibody were significantly decreased (Fig. 6d). Moreover, serum TNF-α, IL-17, and IL-6 levels on day 50 were significantly decreased in mice treated with anti-Sema7A antibody (Fig. 6c).

## Discussion

Although Sema7A has been involved in immunologic disorders, there are few reports regarding its expression and immunological function in RA. The present study is the first, to our knowledge, to show that sSema7A is significantly increased in the synovial fluid and serum of patients with the inflammatory autoimmune disease RA compared with OA. Furthermore, we demonstrate that the levels of sSema7A strongly stimulated inflammatory T-cell and monocyte/macrophage responses in vitro. Second, we demonstrated that Sema7A is involved in the inflammatory response in RA and is also correlated with disease activity. We assessed the relationship between serum sSema7A levels and known clinical and biologic markers usually used in the diagnosis and prediction of RA. Interestingly, a significant correlation between serum sSema7A levels and the levels of DAS28, CRP, and RF in patients with RA was observed. Furthermore, successful treatment with biologic DMARDs in patients with RA decreased serum sSema7A levels. In general, these data indicate that sSema7A is a potentially useful biomarker for RA disease activity, further supporting its role in the progression of RA.

An increasing amount of data shows that Sema7A is an important player during the immune response because it regulates the interactions between T cells and monocytes during the primary immune response [29]. The immunogenetics of RA suggest the key role for abnormal pathways of T-cell and monocyte activation in the perpetuation of disease [46]. Thus, we decided to investigate the role of Sema7A in RA. Recombinant sSema7A was performed to evaluate its effects on T cells and monocytes. Our data reveal that sSema7A regulated cytokine secretion by T cells and monocytes. An analysis of T-cell and monocyte cytokine production showed that stimulation with Sema7A led to strongly enhanced production of proinflammatory cytokines, such as TNF-α and IL-6. Interestingly, it has been shown that these cytokines play central roles in RA disease progression and recruit inflammatory cells to erode the joints of patients with RA [46]. It thus appears that Sema7A is involved in the pathogenesis of RA.

In this study, we also demonstrated that the production of the inflammatory cytokines IFN-γ (formally attributed to Th1 response) and IL-17 (the well-known Th17-derived cytokine) was significantly increased in T-cell culture supernatant induced by sSema7A. These results were also confirmed by the significant upregulation of the expression of transcription factor T-bet and RORγt in CD4^+^ T cells following stimulation with Sema7A. A previous report suggested that T-bet and RORγt promote the differentiation of T cells into Th1/Tc1 and Th17/Tc17 subsets, respectively [47]. Recently, it has become clear that both Th1 and Th17 cytokines are key players in the development of RA [48]. Furthermore, we observed that Sema7A significantly enhanced the production of Th17/Tc17 cytokines, including IFN-γ, IL-22, and IL-17, in comparison with cells treated with DSema7A. Collectively, these findings demonstrate that Sema7A can strongly elevate the production of inflammatory cytokines, particularly Th17/Tc17 and Th1/Tc1, which is strongly similar to the characteristic cytokine profile of patients with RA displayed by T cells in vitro.

To further determine the possible mechanism of action of Sema7A, we explored intracellular signal transduction pathways. Plexin C1 and β1-integrin molecules were shown to serve as Sema7A receptors. To determine whether both receptors may play roles in the stimulatory effect of Sema7A observed in T cells and monocytes, we evaluated the plexin C1 and β1-integrin mRNA levels in T cells and monocytes isolated from patients with RA and healthy subjects. Both receptor mRNA levels were significantly upregulated in patients with RA compared with healthy subjects. Next, we tested whether Sema7A mediates its intracellular effects via interactions with plexin C1 and β1-integrin. Blocking assays were performed in CD14^+^ cells with anti-β1-integrin antibody or anti-plexin C1 antibody in the presence or absence of Sema7A. Interestingly, Sema7A-induced cytokine secretion was attenuated by the blockage of β1-integrin but not plexin C1 in CD14^+^ cells, suggesting that β1-integrin is a binding partner for Sema7A in monocytes. As previously reported, our results showed that the Sema7A-induced cytokine secretion from monocytes was also significantly inhibited by an anti-β1-integrin mAb alone. In the present study, we observed that Sema7A induces the activation of FAK in CD14^+^ cells. These results suggest that Sema7A signaling occurs through the engagement of β1-integrin in monocytes, given that signaling through integrins commonly leads to FAK activation.

In the present study, the expression of Sema7A mRNA was stable in CD4^+^ cells, and CD14^+^ cells. However, the expression levels of Sema7A at the cell surface were actually decreased in T cells and monocytes from the peripheral blood of patients with RA. Thus, we speculated that the relative decrease in cellular Sema7A levels may be attributable to the shedding of Sema7A from the cell surface. In support of this notion, other studies established that the transmembrane semaphorins can be proteolytically cleaved to produce soluble proteins, and the proteinase responsible for cleavage has been identified: It is a member of the ADAM family [49]. It has been shown that the cleavage of GPIbα is mediated by ADAM17. On the basis of our analysis of the results described above, we speculated that Sema7A, a GPI-anchored member of the semaphorin family, might be one of the proteins cleaved by ADAM17. Consistent with this finding, our results validated the detection of Sema7A in the shedding analysis and suggest that ADAM17 was probably the sheddase. In addition, our results indicated that sSema7A induced TNF-α and IL-6 production and that both TNF-α and IL-6 induced the production of ADAM17. The levels of ADAM17 were increased in synovial fluid and serum from patients with RA. Hence, we propose that Sema7A functions as an autocrine stimulator of the IL-6/TNF-α inflammatory axis in patients with RA.

To further confirm that Sema7A has a distinct role in the pathogenesis of RA in vivo, we introduced anti-Sema7A antibody into a T-cell- and monocyte-dependent animal model of RA, namely, CIA. Blocking Sema7A in CIA exerted favorable therapeutic effects and achieved a dramatic arrest in disease progression, as confirmed by the clinical, histopathological, and immunological manifestations of arthritis. These observations extend our findings demonstrating that Sema7A has a distinct role in regulating inflammatory responses in vivo, suggesting that Sema7A might be a possible therapeutic target for RA. Furthermore, sSema7A has been reported to promote angiogenesis. Angiogenesis is thought to have an important role in the progression of arthritic lesions in RA. Therefore, it is attractive to speculate that Sema7A may be further involved in the pathogenesis of RA by promoting the angiogenesis of joint tissue, which would recruit the inflammatory cells into the synovium.

## Conclusions

Taken together, our results show that the levels of secreted Sema7A were significantly high in the serum and synovial fluid of patients with RA and that Sema7A is a promising biomarker for RA disease. The crucial roles of Sema7A in the pathogenesis of RA suggest that Sema7A is a potential therapeutic target for RA. Therefore, our results provide a further rationale for prospective clinical studies designed to evaluate whether Sema7A provides effective therapeutic activities in RA and other autoimmune diseases.

## Abbreviations

ADAM: A disintegrin and metalloprotease
CIA: Collagen-induced arthritis
CII: Collagen type II
CRP: C-reactive protein
DAS28: Disease Activity Score in 28 joints
DMARD: Disease-modifying antirheumatic drug
DSema7A: Heat-denatured Sema7A
ELISA: Enzyme-linked immunosorbent assay
FACS: Fluorescence-activated cell-sorting
FAK: Focal adhesion kinase
GPI: glycosylphosphatidylinositol
IFN-γ: Interferon-gamma
IgG: Immunoglobulin G
IL-17: Interleukin-17
mAb: Monoclonal antibody
mRNA: Messenger RNA
OA: Osteoarthritis
PBMC: Peripheral blood mononuclear cell
PE: phycoerythrin
PFAK: Phosphorylated focal adhesion kinase
RA: Rheumatoid arthritis
RF: Rheumatoid factor
RORγt: Retinoic acid receptor-related orphan nuclear receptor γt
RT-PCR: Reverse transcription-polymerase chain reaction
Sema7A: Semaphorin 7A
shRNA: Short-hairpin RNA
sSema7A: Soluble semaphorin 7A
Th17: T-helper cell type 17
TNF-α: Tumor necrosis factor-α
